# SIRT1 stabilizes extrachromosomal gene amplification and contributes to repeat-induced gene silencing

**DOI:** 10.1016/j.jbc.2021.100356

**Published:** 2021-02-02

**Authors:** Ryonosuke Taniguchi, Koichi Utani, Bhushan Thakur, Kazuho Ishine, Mirit I. Aladjem, Noriaki Shimizu

**Affiliations:** 1 Graduate School of Biosphere Science, Hiroshima University, Higashi-Hiroshima, Hiroshima, Japan; 2 Laboratory of Molecular Pharmacology, Center for Cancer Research, National Cancer Institute, National Institutes of Health, Bethesda, Maryland, USA; 3 Graduate School of Integrated Sciences for Life, Hiroshima University, Higashi-Hiroshima, Hiroshima, Japan

**Keywords:** gene amplification, sirtuin1, repeat-induced gene silencing, extrachromosomal element, gene expression, histone deacetylase, protein expression, genomic instability, BFB, breakage-fusion-bridge, *d2EGFP*, destabilized enhanced GFP, DIG, digoxigenin, DM, double minute, DSB, double strand breakage, FISH, fluorescence *in situ* hybridization, HDAC, histone deacetylase, HSR, homogeneously staining region, MAR, matrix attachment region, RIGS, repeat-induced gene silencing, SIRT1, Sirtuin 1

## Abstract

Sirtuin 1 (SIRT1) is a protein deacetylase that maintains genome stability by preventing the activation of latent replication origins. Amplified genes in cancer cells localize on either extrachromosomal double minutes (DMs) or the chromosomal homogeneously staining region. Previously, we found that a plasmid with a mammalian replication initiation region and a matrix attachment region spontaneously mimics gene amplification in cultured animal cells and efficiently generates DMs and/or an homogeneously staining region. Here, we addressed the possibility that SIRT1 might be involved in initiation region/matrix attachment region–mediated gene amplification using SIRT1-knockout human COLO 320DM cells. Consequently, we found that extrachromosomal amplification was infrequent in SIRT1-deficient cells, suggesting that DNA breakage caused by latent origin activation prevented the formation of stable extrachromosomal amplicons. Moreover, we serendipitously found that reporter gene expression from the amplified repeats, which is commonly silenced by repeat-induced gene silencing (RIGS) in SIRT1-proficient cells, was strikingly higher in SIRT1-deficient cells, especially in the culture treated with the histone deacetylase inhibitor butyrate. Compared with the SIRT1-proficient cells, the gene expression per copy was up to thousand-fold higher in the sorter-isolated highest 10% cells among the SIRT1-deficient cells. These observations suggest that SIRT1 depletion alleviates RIGS. Thus, SIRT1 may stabilize extrachromosomal amplicons and facilitate RIGS. This result could have implications in cancer malignancy and protein expression.

Gene amplification plays a pivotal role in tumor progression by providing cells with growth advantages or drug resistance. Highly amplified genes localize either at the extrachromosomal, circular, and autonomously replicating double minutes (DMs) or at the chromosomal homogeneously staining region (HSR). The location of amplified genes in human cancer is closely associated with disease prognosis (1). DMs and the HSR often share the same sequence (2), and mutual conversions between them are correlated with the development of therapy resistance in human cancer (3, 4). Amplified genes are more frequently localized at DMs than in the HSR in *in vivo* tumor tissues (5). Extrachromosomal amplification at DMs or episomes drives tumor evolution and genetic heterogeneity (6, 7). Conversely, a decrease in the number of amplified genes at DMs is associated with growth arrest, cellular differentiation (8, 9), or loss of tumorigenicity (10). Such de-amplification occurs spontaneously (8) or is induced by low concentrations of replication inhibitors such as hydroxyurea (11) and is accompanied by the selective entrapment of DMs in cytoplasmic micronuclei (9, 10, 12). Previously, we have shown that random double strand breakage (DSB), caused by low concentrations of hydroxyurea (13), or targeted DSB at the DMs (14) induces the aggregation of multiple DMs in the nucleus, which generates micronuclei composed of DMs after mitosis.

In addition, we have found that a plasmid with a mammalian replication initiation region (IR) and a matrix attachment region (MAR) initiates gene amplification in cultured mammalian cells and efficiently generates DMs or HSRs (15, 16). This plasmid mimics the episome model of gene amplification (17, 18) in cancer cells. The involvement of extrachromosomal circular DNA in gene amplification was recently reported in plant herbicide resistance (19). The IR/MAR plasmid is multimerized during its autonomous replication to a tandem repeat and is maintained at the extrachromosomal site (16, 20). Such multimers might be integrated into the chromosome arm and induce breakage-fusion-bridge (BFB) cycles that elongate the amplified regions to generate long HSRs (20, 21). The amplification of a gene of interest could be targeted to a mammalian artificial chromosome (22). The IR/MAR gene amplification was useful for basic chromosome biology (reviewed in [23]) as well as for recombinant protein production (24, 25). However, the amplified genes are frequently subjected to epigenetic gene silencing (26), by a mechanism known as repeat-induced gene silencing (RIGS) (27). RIGS is an important biological process that heterochromatinizes pericentromeric regions to establish their mechanical strength (28), silences transposons to prevent their spread (29), or silences transgenes (30, 31).

Sirtuin 1 (SIRT1) is an NAD-dependent deacetylase involved in many biological processes (reviewed in [32, 33, 34]). Recently, we found that SIRT1 preserves genomic stability by preventing the activation of latent origins of replication (35), suggesting that SIRT1 might affect gene amplification in cancer cells. Therefore, in the current study, we examined the effect of SIRT1 deficiency on gene amplification at DMs or HSRs using our original IR/MAR gene amplification system. Consequently, we found that SIRT1 is involved in extrachromosomal element stability and serendipitously uncovered the involvement of SIRT1 in RIGS.

## Results

### SIRT1-KO cells demonstrated infrequent extrachromosomal gene amplification of the IR/MAR plasmid

**Figure 1:**
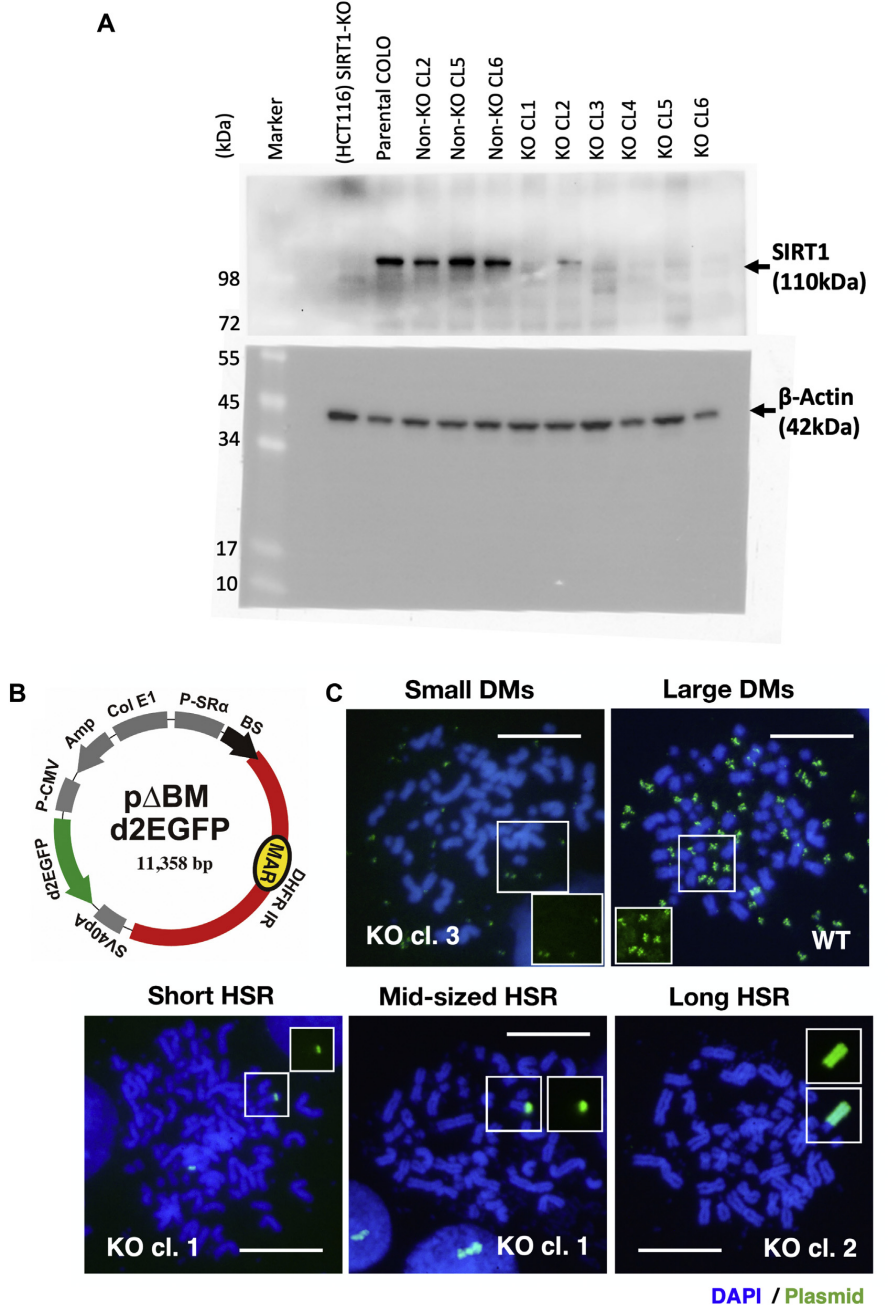
**Establishment of SIRT1-KO COLO 320DM clones and experimental set-up.***A*, *SIRT1* was disrupted in COLO 320DM cells using CRIPR/Cas9 and SIRT1 KO clones were identified by Western blotting with a rabbit monoclonal antibody ab156585 (Abcam) using beta-actin (antibody A5316, Sigma Aldrich) as a loading control. *B*, structure of plasmid pΔDBM d2EGFP, which was used throughout this study. *C*, representative FISH images showing amplification of the IR/MAR plasmid. The hybridized plasmid probe was detected as *green fluorescence* within the DAPI-stained metaphase chromosome spread (*blue*). Images for cells with large and small DMs and short, mid-sized, and long HSRs are shown. The amplified region detected by FISH was surrounded by *square*, and single-color FISH image of the region is inserted to each image. Scale bars: 10 μm. DAPI, 4′,6-diamidino-2-phenylindole; DM, double minutes; FISH, fluorescence in situ hybridization; HSR, homogeneously staining region; IR, initiation region; MAR, matrix attachment region.

We successfully depleted the *SIRT1* gene in human colorectal carcinoma COLO 320DM cells using CRISPR/Cas9 and examined SIRT1 protein expression in isolated clonal cells by Western blotting. Consequently, we obtained several clones (KO clones) that exhibited complete depletion of the SIRT1 protein (Fig. 1*A*). These KO-cells showed almost similar growth rate to the wildtype (WT) cells.

**Figure 2:**
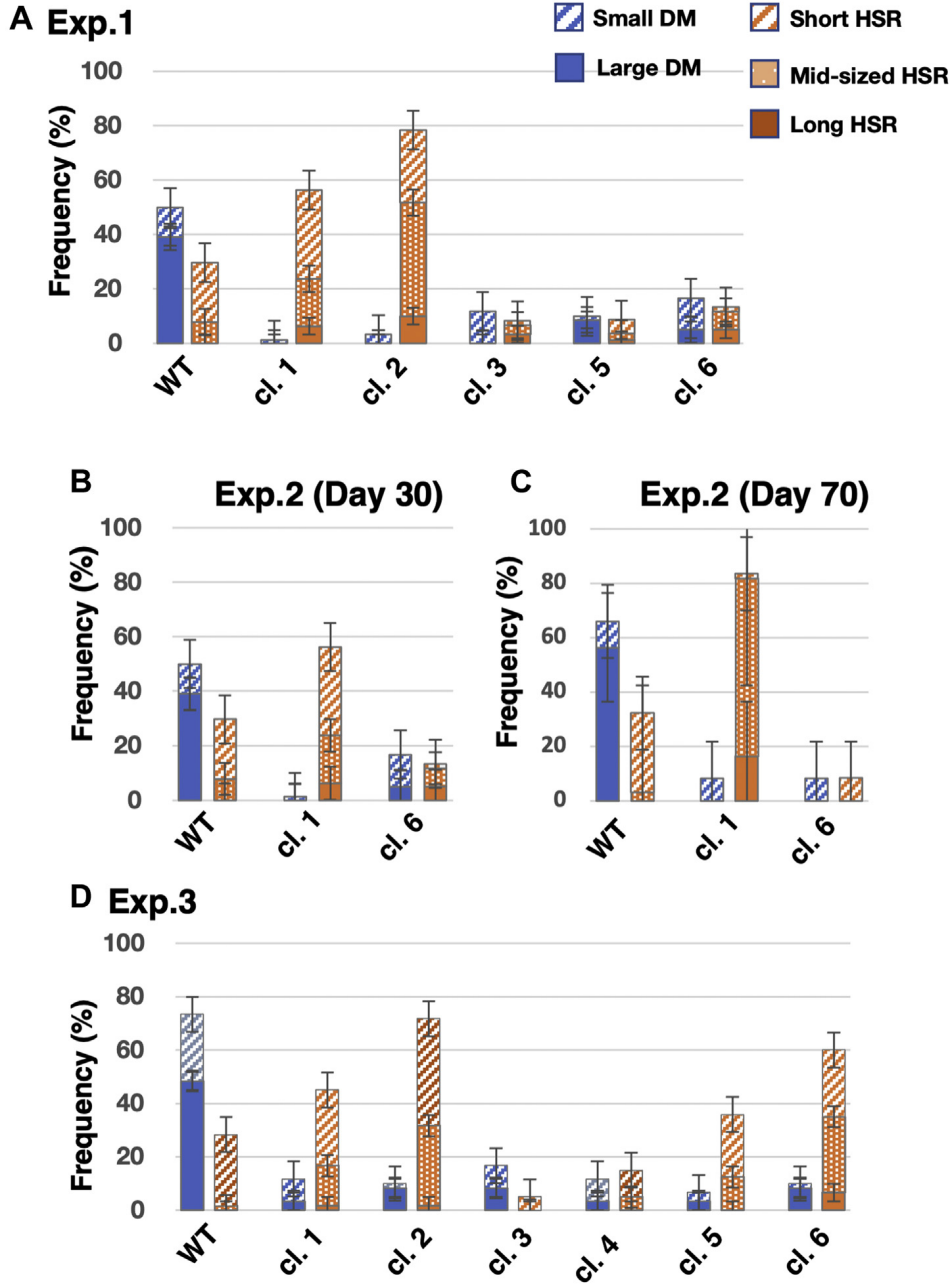
**The IR/MAR plasmid was less efficiently amplified at the extrachromosomal DMs in SIRT1 KO cells.** Plasmid pΔBM d2EGFP was transfected into WT COLO 320DM cells or SIRT KO clone 1 to 6 cells; stable transformants were selected on blasticidin for ca. 30 days. The chromosome spread was analyzed by FISH using a plasmid probe; representative images are shown in Figure 1*C*. The frequency of cells bearing these structures was determined by examining >20 metaphase cells in triplicate for each slide and the mean ± SD was plotted. The transfection, selection, and FISH examination were independently conducted three times and are designated as Exp. 1 (*A*), Exp. 2 (*B*, *C*), and Exp. 3 (*D*). DMs, double minutes; FISH, fluorescence *in situ* hybridization; IR, initiation region; MAR, matrix attachment region.

We transfected an IR/MAR-bearing plasmid (pΔBM d2EGFP; Fig. 1*B*) into WT or SIRT1 KO cell clones, selected stable transformants on blasticidine for approximately 1 month, prepared chromosome spreads, and conducted fluorescence *in situ* hybridization (FISH) analysis to detect the plasmid sequences. The plasmid demonstrated amplification at large and small DMs and short, mid-sized, or long HSRs (the representative images, see Fig. 1*C*), as observed in our previous studies (15, 16). As previously observed, the amplified IR/MAR plasmid at DMs or HSR in a single metaphase is mutually exclusive in most cells (15, 16). Therefore, we next measured the frequencies of the various sizes of DMs and HSRs in three independent transfections, selections, and FISH analyses (Exp. 1∼3; Fig. 2). Compared with WT cells, we found that plasmid amplification at the DMs was consistently inefficient in six independent SIRT1 KO clones in three independent experiments. Amplification at the chromosomal HSR varied between the different KO clones; however, the clonal variation was reproducible among three independent experiments, and the relative frequency of DMs and HSRs remained consistent 30 to 70 days posttransfection (Fig. 2, *B* and *C*).

### Amplified genes generated in the SIRT1 KO COLO 320DM cells showed higher gene expression

**Figure 3:**
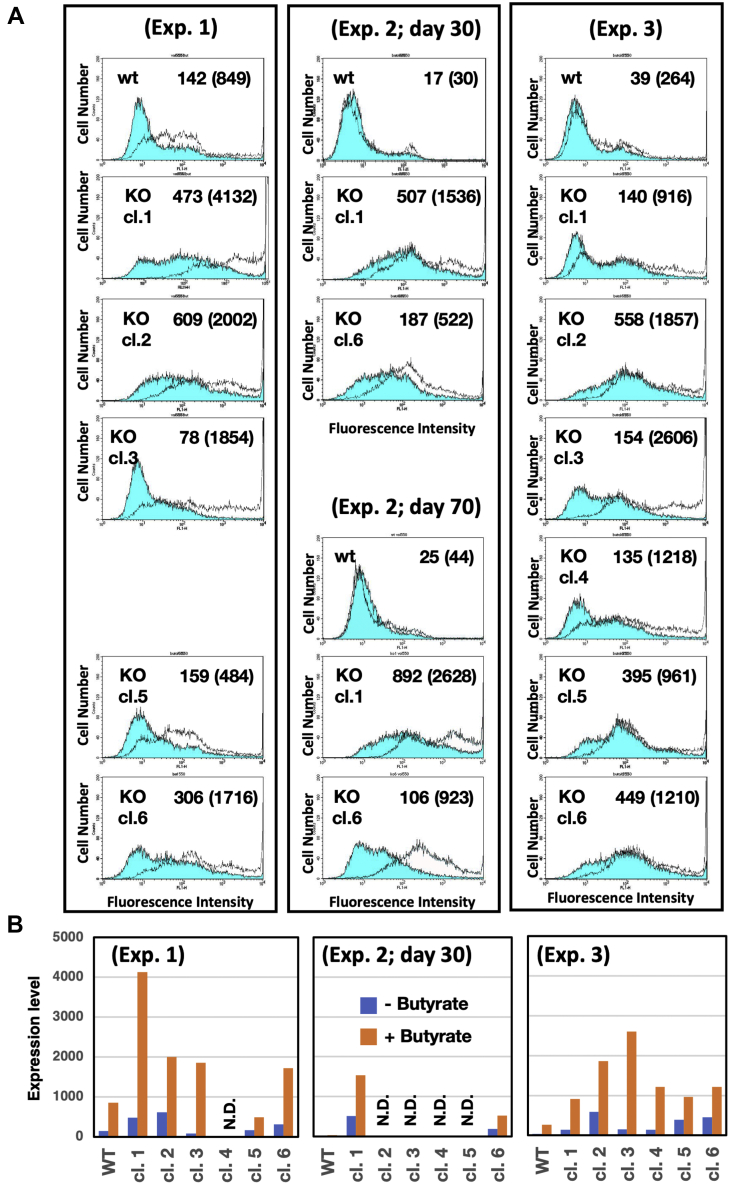
**SIRT1 deficiency and butyrate treatment synergistically enhanced gene expression from the amplified sequences.***A*, *d2EGFP* expression in the transformants shown in Figure 2 was analyzed by flow cytometry in the absence (*blue*-*filled lines*) or presence (*unfilled lines*) of 2 mM sodium butyrate during the last 3 days. The mean fluorescence intensity with and without butyrate (*in parentheses*) is indicated in each chart. *B*, the expression is summarized graphically. *d2EGFP*, destabilized enhanced GFP; N.D.; not done; SIRT1, sirtuin 1.

As the pΔBM d2EGFP plasmid contains a destabilized enhanced GFP (*d2EGFP*) expression cassette, we were able to measure its expression by flow cytometry (Fig. 3). The *d2EGFP* contained PEST domain that destabilize the protein product; therefore, it is adequate for evaluating the gene expression in real-time. Unexpectedly, we found that the expression of the EGFP marker was much higher in SIRT1 KO cells than in WT cells. This result was consistently obtained from different SIRT1 KO clones and was reproducible among three independent transfections and selections (Exp. 1–3). We also analyzed the cells treated with butyrate, because the drug augmented gene expression from the amplified transgene (25, 36). The high expression in the SIRT1 KO cells was further enhanced by treatment with butyrate, an inhibitor of class I and class II histone deacetylase complexes but not sirtuins (Fig. 3). These results indicated that the effects of SIRT1 deficiency and butyrate treatment were synergistic.

**Figure 4:**
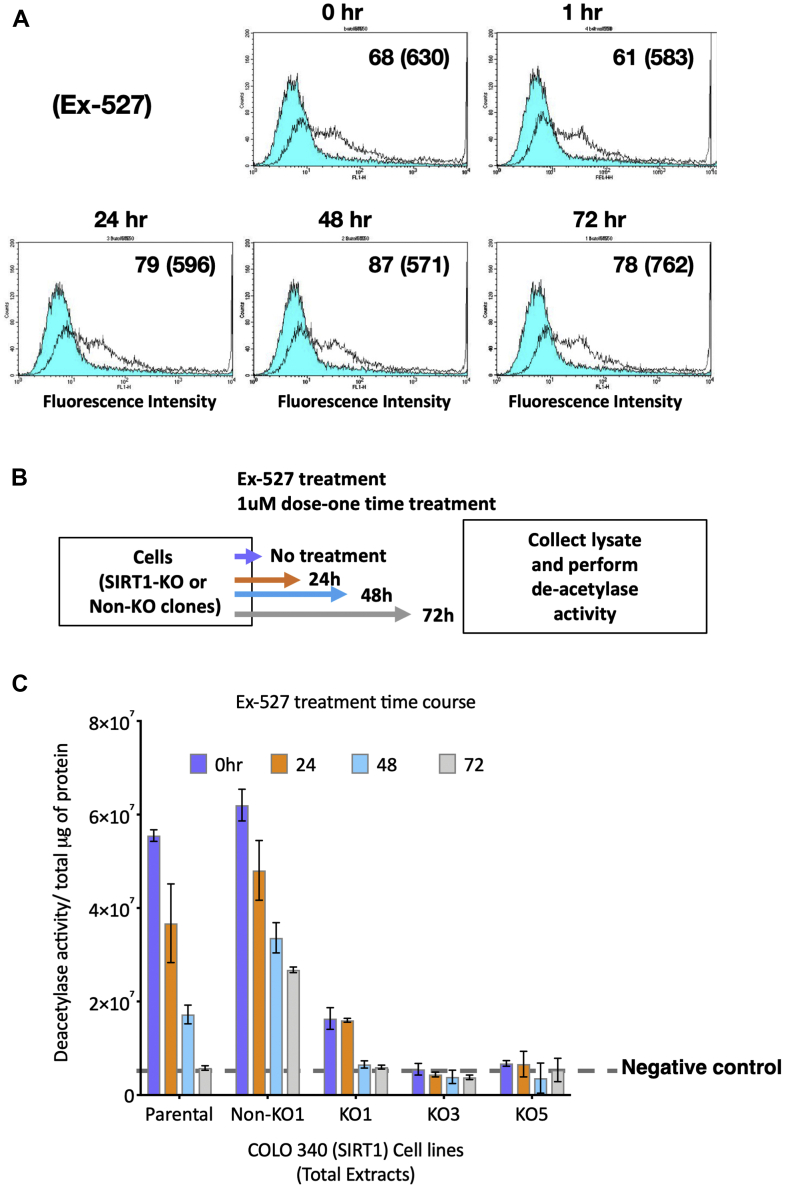
**Transient inhibition of SIRT1 did not increase the expression of amplified genes.***A*, the transformants from WT COLO 320DM cells in Exp. 1 were cultured in the presence of 2 μM Ex-527, a specific inhibitor of SIRT1, for the indicated time and were then analyzed by the flow cytometry in the absence (*blue-filled lines*) or presence (*unfilled lines*) of 2 mM sodium butyrate during the last 3 days. The mean fluorescence intensity with and without butyrate (*in parentheses*) is indicated in each chart. *B*, strategy for measurement of SIRT1 de-acetylation activity. *C*, cells were cultured in presence of 1 μM EX-527 for 24 to 72 h and SIRT1 de-acetylation activity was measured from total cell extracts as fluorescence intensity and normalized with protein level. SIRT1, sirtuin 1.

We next examined whether transient inhibition of SIRT1 activity by a specific inhibitor could also alleviate the suppression of genes expressed in the amplified sequences in SIRT1-proficient cells by adding EX-527 to stable transformants from WT cells (Exp. 1) and examining d2EGFP expression (Fig. 4*A*) and deacetylation activity (Fig. 4, *B* and *C*). EX-527 was highly stable in the culture medium as a single dose of EX-527 markedly inhibited the deacetylation activity of SIRT1 after 72 h. Because a 72-h exposure to the inhibitor did not elevate gene expression from the amplified sequences (Fig. 4*A*), we conclude that SIRT1 participates in the establishment of silencing during the gene amplification process and is not required for the maintenance of the silenced phenotype.

### Gene expression/copy was extremely high in the SIRT1 KO cells

**Figure 5:**
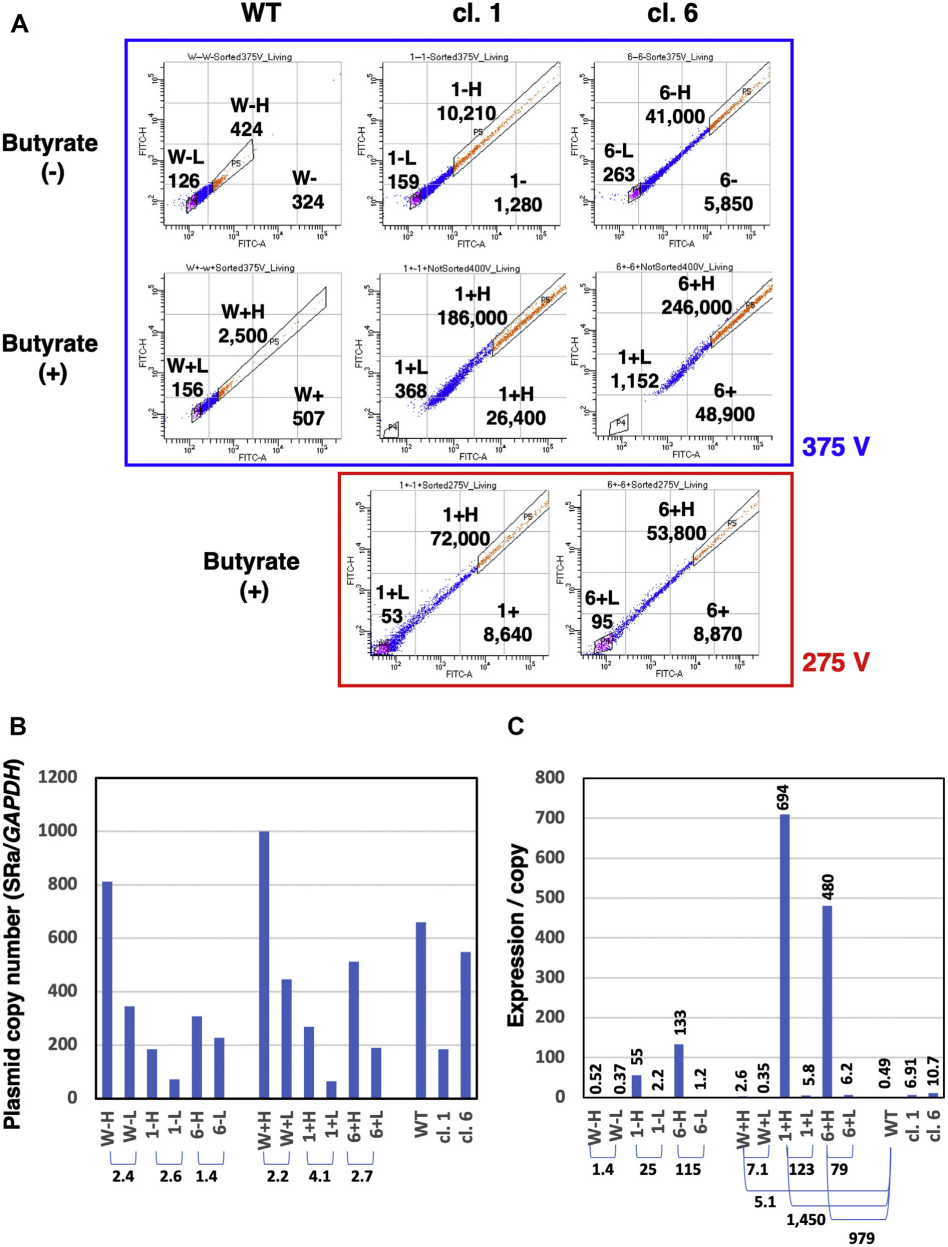
**Gene expression/copy was extremely high in SIRT1 KO cells**. *A*, the transformants from WT, KO cl. 1 and cl. 6 in Exp. 3 (Figs. 2*D* and 3) were cultured in the absence (−) or presence (+) of butyrate for 3 days, and cells with the highest 10% (H; *red dots*) and lowest 10% (L; *red dots*) GFP fluorescence intensities were segregated using a cell sorter. The analyses and sorting was performed by using a laser strength of 375 V for most of the cell populations; however, it was lowered to 275 V for butyrate-treated KO cl. 1 and 6 because a significant number of these cells were scaled out under 375 V. The mean fluorescence intensity of the entire population, and the highest 10% or the lowest 10% cells are mentioned in each chart. *B*, Total DNA was subject to real-time PCR to determine the copy number of plasmid sequence (*SRa* promoter) compared with endogenous *GAPDH*. The ratio between H and L populations is also mentioned below the graph. *C*, the gene expression (mean fluorescence intensity in *A*) per gene copy (*B*) was calculated and is shown in the graph. The ratios between the indicated pair are noted below the graph. SIRT1, Sirtuin 1.

The transformants, which were generated from wild-type, KO clone 1 and clone 6 in Exp. 3 (Figs. 2*D* and 3), were cultured in the absence (−) or presence (+) of butyrate, and cells with the highest (H; 10%) and lowest (L; 10%) GFP fluorescence intensities were segregated using a cell sorter (Fig. 5*A*). Again, GFP expression was much higher in SIRT1 KO clone 1 and 6 compared with the WT cells, and the expression was further increased by butyrate treatment. The average expression of total, H, or L cells was noted in each chart.

Total DNA was isolated from the sorted cells and subject to real-time PCR to determine the plasmid copy number (SRα promoter) relative to endogenous *GAPDH*. The result (Fig. 5*B*) showed that the copy number was constantly higher in the highest expression cells compared with the lowest expression cells, and the ratio between them was in the range of 1.4 (6−) to 4.1 (1+), suggesting that the gene dosage effect may have partly contributed to the higher expression. We then divided the gene expression value (mean fluorescence intensity) by the gene copy number to determine the level of expression per copy number (Fig. 5*C*). Consequently, we found that *d2EGFP* expression levels per copy were extraordinarily high in the higher-expressing fraction from the SIRT1 KO clone. For example, as shown in Figure 5*C*, SIRT1 KO populations showed notably high ratios of *d2EGFP* expression values (25 and 115 for −, and 123 and 79 for +) compared with the WT cell population (1.4 for – and 7.1 for +). Consequently, butyrate-treated cells with high *d2EGFP* expression showed 1450-fold (KO clone 1) or 979-fold (KO clone 6) higher expression/copy compared with the control unsorted WT cells. These observations demonstrated that the population of SIRT1 KO cells contained cells showing extraordinarily high expression/copy, suggesting that the depletion of SIRT1 resulted in alleviation from repeat-induced gene silencing.

### Stable clones showing considerably higher gene expression were efficiently isolated

**Figure 6:**
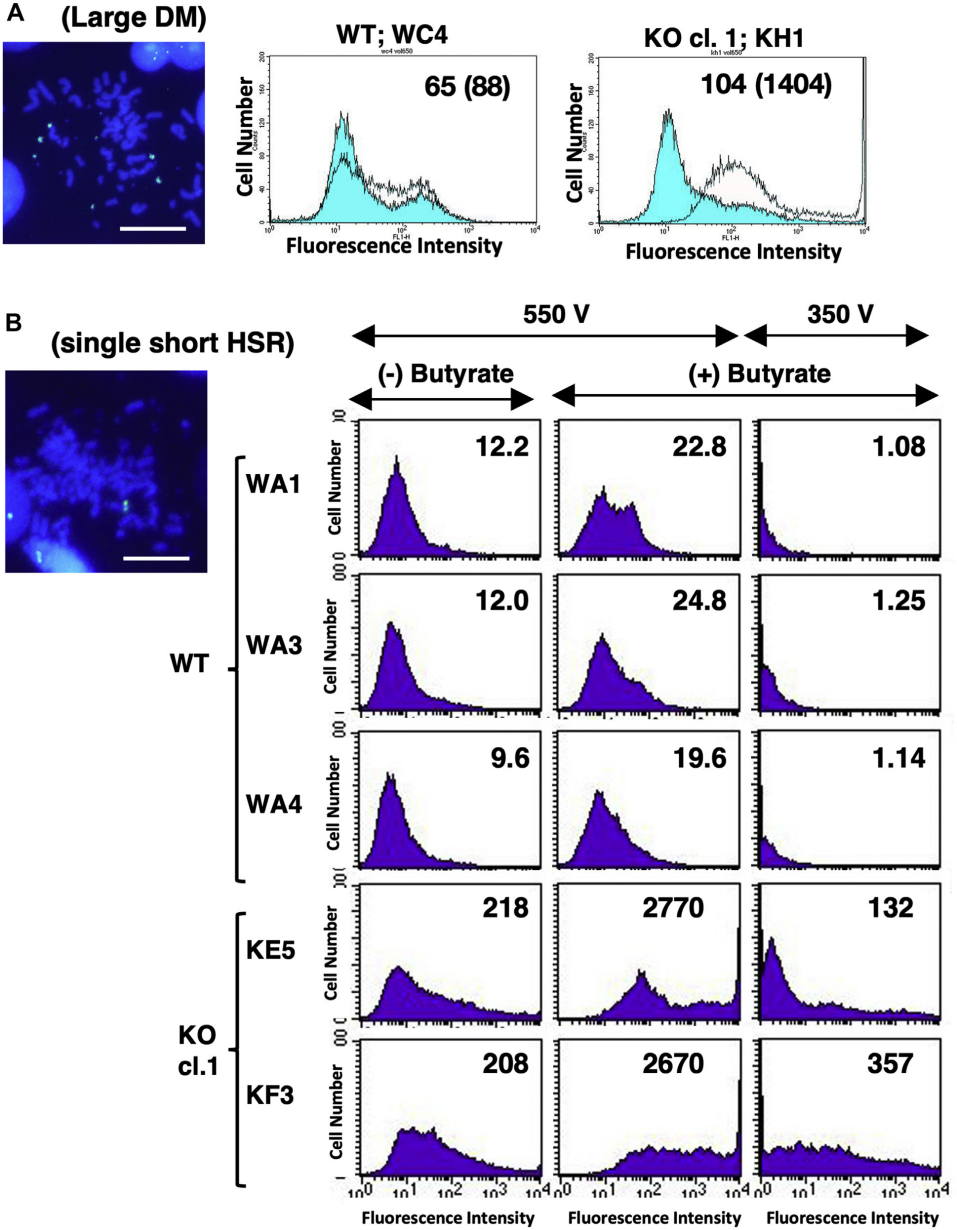
**Stable clones showing quite high gene expression were efficiently isolated**. More than 15 subclones were isolated from each WT or SIRT1 KO clone 1 transformants in Exp. 1 and examined by FISH. According to FISH results, we selected subclone WC4 and KH1 from WT and KO clone 1, respectively, as semipure subclones that had similar large DMs with a similar number/cell (*A*). According to FISH results, we also selected subclone WA1, WA3, and WA4 from WT, and KE5 and KF3 from KO clone 1, as semipure subclones that had a similar short HSR. Scale bars: 10 μm. These clonal cells were cultured in the absence (*unfilled lines* in *A*; indicated in *B*) or presence (*blue*-*filled lines* in *A*; indicated in *B*) of 2 mM butyrate and analyzed by flow cytometry. In (*B*), because the expression was too high for accurate measurement, the laser voltage was lowered, as indicated, for the butyrate-treated culture. DMs, double minutes; FISH, fluorescence in situ hybridization; HSR, homogeneously staining region; SIRT1, sirtuin 1.

To address the question whether higher gene expression in SIRT1-deficient cells was stable or not during culturing, we isolated clones from WT and KO cl. 1 transformants (Exp. 1) and examined them by both FISH and flow cytometry. After careful examination by FISH, we identified several clones bearing similar amplifications at large DMs (Fig. 6*A*) or similar, single short HSRs (Fig. 6*B*). Flow cytometric examination of d2EGFP expression in these clones revealed that the expression was constantly and substantially higher in SIRT1 KO cells than in WT cells. This result was obtained for the amplicons residing in both DMs or HSR. Furthermore, this effect was more pronounced when we compared the cultures treated with butyrate, further suggesting that SIRT1 deficiency and butyrate treatment have a synergistic effect. We concluded that gene expression per amplified gene copy was higher in SIRT1 KO cells, and the phenotype was stable, thus enabling the efficient isolation of a stable cell line that expresses a recombinant protein at a considerably high level.

## Discussion

**Figure 7:**
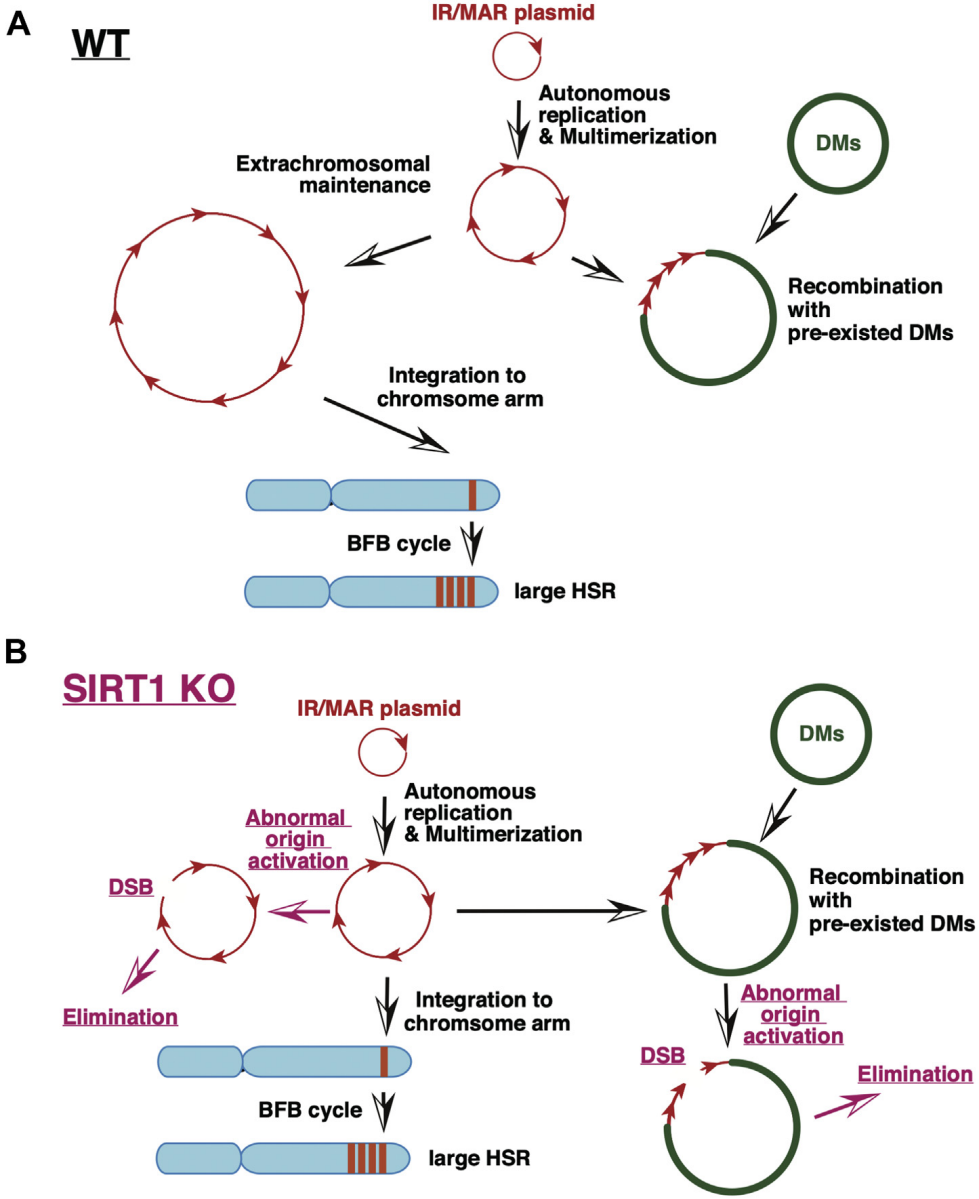
**A model for gene amplification in wildtype (WT; *A*) and SIRT1 KO (*B*) cells**. BFB, breakage-fusion-bridge; DMs, double minutes; DSB, double strand breakage; HSR, homogeneously staining region; SIRT1, sirtuin 1.

Here, we have shown that amplification at the extrachromosomal DMs of the IR/MAR plasmid was inefficient in SIRT1-deficient cells (Fig. 2). Our previous studies have revealed that the IR/MAR plasmid is initially amplified to a direct repeat at the extrachromosomal context, and the tandem repeat is then integrated into the chromosome arm, where it is elongated to a large HSR by efficiently inducing the BFB cycle (Fig. 7*A*, [16, 20]). Notably, we have also shown that SIRT1 prevents dormant origin activation, thereby preserving genome stability (35). Therefore, SIRT1 deficiency would likely result in DSB among the tandem repeats of IR/MAR sequences that potentially function as replication origins (37, 38). In addition, we have shown that DSB in the extrachromosomal DMs, which was induced by low concentrations of replication inhibitors or directly induced by CRISPR/Cas9, was sufficient to aggregate multiple DMs in the nucleus and eliminate them (13, 14). Therefore, the tandem IR/MAR plasmid multimer would suffer from frequent DSB and be eliminated during amplification in SIRT1-deficient cells (Fig. 7*B*). In contrast, the generation of HSRs requires the induction of BFB cycles. The BFB cycle is induced by DSB within the plasmid repeat in the chromosomal context, the efficiency of which would depend on plasmid repeat length. Therefore, differences in the extent of extrachromosomal multimerization between the SIRT1 KO clones would differently trigger the BFB cycle, thus generating variations in HSR generation between the SIRT1 KO clones (Figs. 2 and 7*B*).

The predicted function of SIRT1 as a transcriptional silencer is based on its yeast ortholog, SIR2, which plays a role as a silencer and a histone deacetylase (HDAC) (39). In mammals, SIRT1 prevents tumorigenic transformation by epigenetically silencing cancer-promoting genes (40) or leukemic genes (41). SIRT1-mediated silencing can also sensitize cells to TNFalfa-induced apoptosis (42) and promote autophagy (43). Here, we serendipitously found that the genes amplified in SIRT1-deficient cells exhibited much higher expression (Figure 3, Figure 4, Figure 5, Figure 6). SIRT1 is a class III HDAC, whereas butyrate is an inhibitor of class I and IIa HDACs (44). Histone acetylation is generally associated with higher gene expression; therefore, HDAC inhibition would generate a relaxed chromatin with higher gene expression. SIRT1-deficiency and the butyrate treatment synergistically augmented the expression of the amplified genes, because these conditions inhibit different classes of HDACs. Therefore, their combination will be highly useful for recombinant protein production.

Genes amplified by the IR/MAR method are frequently subject to RIGS. As mentioned in the Introduction, RIGS has an important cellular function as it establishes the mechanical strength of centromeres, prevents transposon spreading, and silences foreign transgenes. In this study, we found that RIGS was at least partially alleviated in the genes amplified in SIRT1-deficient cells; in other words, SIRT1 is involved in the establishment of RIGS. Because SIRT1 inhibition did not alleviate gene silencing, our observations also imply that once RIGS is established, SIRT1 activity is no longer required for the maintenance of the silenced state.

SIRT1 could either promote or inhibit cancer progression, depending on the specific molecular target or cancer type (45). Our current study suggests that SIRT1 stabilizes the extrachromosomal amplification structures, which are critical to the progression of many human malignancies. Furthermore, SIRT1 plays a role in genome maintenance through RIGS, which establishes centromere structure and repeat stability. Therefore, our present study discovered new roles of SIRT1 in genome stability by modulating extrachromosomal gene amplification and RIGS, potentially leading to important implications in cancer malignancy and protein expression.

## Experimental procedures

### Cell culture and drugs

The human colorectal carcinoma COLO 320DM (CCL-220) cell line was originally derived from the American Type Culture Collection (ATCC), and clone “COLO 320DM #3” bearing multiple DMs (15) was used in this study. The cells were cultured in RPMI 1640 medium supplemented with 10% fetal bovine serum (Biowest). Hamster cell line CHO K1 (CCL 61) was obtained from the Cell Research Center for Biomedical Research, Institute of Development, Aging, and Cancer, Tohoku University, Japan. Hamster CHO DG44 cells were obtained from Dr Lawrence Chasin at Columbia University. The cells were grown in Ham's F-12 supplemented with 10% fetal bovine serum (EuroClone) and maintained using TrypLE Express with Phenol Red (12605-028; Invitrogen). Ex-527 (Merck) was dissolved in DMSO at 100 mM and added to the culture at a final concentration of 2 μM.

### Establishment of SIRT1 knockout COLO 320DM cell clones

We established SIRT1-depleted cell lines from COLO 320DM cells as previously described (35) with modification. Plasmid pX330-U6-Chimeric_BB-CBh-hSpCas9 was a gift from Feng Zhang (Addgene plasmid #42230; https://www.addgene.org/42230/; RRID; Addgene 42230). The guide RNA sequence for the human *SIRT1* gene (NM_012238.5) was designed with CRISPR design tool DNA2.0 (https://www.atum.bio/eCommerce/cas9/input). The sg-oligo sequence was as follows: Cas9 human SIRT1(67–87): GAGGCCGCGTCGTCCCCCGCCGG. The underlined sequence represents the protospacer adjacent motif sequence. The sg-oligo sequence targets exon 1 of human *SIRT1*. We inserted synthetic double-stranded oligonucleotides, which contained the abovementioned sg-oligo sequences, into the *Bbs*I site of pX330-U6-Chimeric_BB-CBh-hSpCas9. Next, we co-transfected the pSpCas9(67–87) and pCR2.1 plasmid bearing a puromycin resistance gene into COLO 320DM (#3) cells using Lipofectamine 2000 (11668019; Invitrogen). The transfected cells were selected on 1 μg/ml puromycin to obtain stable *SIRT1*-deficient cells. From these cells, we isolated clones and checked SIRT1 protein expression by Western blotting using a rabbit polyclonal anti-SIRT1 antibody (07–131; Millipore) or a rabbit monoclonal anti-SIRT1 (p530) antibody (ab156585). These cell clones as well as plasmid used in this study will be deposited to RIKEN Bioresource Center (BRC), Japan, and will be available upon request to BRC.

### Transfection of IR/MAR plasmid

pΔBM d2EGFP was constructed as previously described (46). It contains a *BSR* gene driven by SRαpromoter, the *d2EGFP* gene driven by a CMV promoter, and a *DHFR* IR (*Ori β*; 4634 bp) that contains a sequence showing *in vitro* MAR activity (15). The plasmids were cloned into *Escherichia coli* DH5α cells. Plasmid DNA was purified using the PureLink HiPure plasmid midiprep kit (Invitrogen).

COLO 320DM cells were transfected using GenePORTER2 Transfection Reagent (Genlantis Co), whereas CHO DG44 cells were transfected using Lipofectamine 2000 Reagent (ThermoFisher Scientific Co) according to the manufacturers' recommended protocols. Stable transformants were selected on blasticidin (Funakoshi) or puromycin (Nacalai Tesque).

### FISH and flow cytometry

The metaphase chromosome spread was prepared according to a standard protocol, which involved hypotonic swelling in 75 mM KCl, at 37 °C for 10 min, the centrifugal washing by methanol/acetic acids = 3/1 for three times, followed by dropping onto the prewet slide glass (superfrost, Matsunami Glass Ind. Ltd). The digoxigenin (DIG)-labeled probe for detecting the amplified plasmid sequence was prepared from pΔBM d2EGFP plasmid DNA using the BioPrime DNA labeling system (Invitrogen) combined with 10× DIG DNA labeling mixture (Roche Diagnostics). The hybridized DIG probe was detected with an anti-DIG fluorescein Fab fragment (Roche Diagnostics). The slide was counterstained with 4′,6-diamidino-2-phenylindole and examined using an epifluorescence microscope (TE2000E, Nikon) equipped with a 100× objective lens (Nikon Plan Fluor, NA 1.30 oil) and an appropriate filter set. Digital images were acquired with a Nikon D7000 digital camera and processed with Adobe Photoshop CS6 (Adobe Systems, Inc). For flow cytometry analysis of *d2EGFP* expression, the cells were resuspended in phosphate-buffered saline and analyzed using a FACSCalibur system (Becton Dickinson Co) in the absence or the presence of 2 mM sodium butyrate for the last 3 days of culture.

### Cell sorter and real-time PCR

For isolating cells according to green fluorescence, which reflects the expression of *d2EGFP* in the plasmid, we used FACSAria III (Becton Dickinson) and appropriate setting for FITC because the excitation/emission spectrum of FITC is close to the one for *d2EGFP*. The highest 10% or the lowest 10% cells based on FITC-area (FITC-A) and FITC-height (FITV-H) were sorted directly to the culture medium. A cell concentration of 5 × 10^4^ for each fraction was obtained and collected by centrifugation. Total DNA was extracted based on a published protocol using Triton X100 and proteinase K (8). Real-time PCR was performed on a StepOnePlus system (Applied Biosystems) with Thunderbird qPCR Mix (Toyobo) and gene-specific primers for human genomic *GAPDH* (forward primer: TACTAGCGGTTTTACGGGCG and reverse primer: TCGAACAGGAGGAGCAGAGAGCGA) or SRα promoter that drives *BSR* in the plasmid (forward primer: CTCGCATCTCTCCTTCACG and reverse primer: CGGTCTCGACCTGAGCTTTA). The reaction was performed in triplicate, and their mean values were used to evaluate the copy number of the SRα promoter relative to GAPDH by the ^ΔΔ^Ct method.

### SIRT1 de-acetylase activity measurement

SIRT1 deacetylation activity was measured using a SIRT1 deacetylation kit (Abcam, ab156065) as per manufactures instructions. Briefly total protein from cell were prepared using NP40 buffer (50 mM Tris, pH 7.4, 250 mM NaCl, 5 mM EDTA, 1%NP40) and were mixed with reaction components and fluorometric substrate. This reaction was incubated for 30 min at 37 °C, the fluorometric signal was measured by Spectra Max GEMINIXS (Molecular Devices). The NAD+ samples fluorometric values were normalized by subtracting the control values. Trichostatin A was used to inhibit class I and II HDAC activity.

## Data availability

All data are provided in the manuscript.

## Conflict of interest

The authors declare that they have no conflicts of interest with the contents of this article.
